# Perhexiline maleate in the treatment of fibrodysplasia ossificans progressiva: an open-labeled clinical trial

**DOI:** 10.1186/1750-1172-8-163

**Published:** 2013-10-16

**Authors:** Hiroshi Kitoh, Masataka Achiwa, Hiroshi Kaneko, Kenichi Mishima, Masaki Matsushita, Izumi Kadono, John D Horowitz, Benedetta C Sallustio, Kinji Ohno, Naoki Ishiguro

**Affiliations:** 1 Department of Orthopaedic Surgery, Nagoya University Graduate School of Medicine, 65 Tsurumai, Showa-ku, Nagoya, Aichi 466-8550, Japan; 2 Department of Radiological Technology, Nagoya University Graduate School of Medicine, 65 Tsurumai, Showa-ku, Nagoya, Aichi 466-8550, Japan; 3 Department of Rehabilitation, Nagoya University Graduate School of Medicine, 65 Tsurumai, Showa-ku, Nagoya, Aichi 466-8550, Japan; 4 Department of Cardiology and Clinical Pharmacology, Queen Elizabeth Hospital, 28 Woodville Road, Woodville, SA 5011, Australia; 5 Division of Neurogenetics, Center for Neurological Diseases and Cancer, Nagoya University Graduate School of Medicine, 65 Tsurumai, Showa-ku, Nagoya, Aichi 466-8550, Japan

**Keywords:** Fibrodysplasia ossificans progressiva, Perhexiline maleate, Clinical trial, Biomarker, Whole body CT

## Abstract

**Background:**

Currently, there are no effective medical treatment options to prevent the formation of heterotopic bones in fibrodysplasia ossificans progressiva (FOP). By the drug repositioning strategy, we confirmed that perhexiline maleate (Pex) potentially ameliorates heterotopic ossification in model cells and mice. Here, we conducted a prospective study to assess the efficacy and safety of Pex in the treatment of FOP patients.

**Methods:**

FOP patients in this open-label single-center study were treated with Pex for a total of 12 months, and followed up for 12 consecutive months after medication discontinuation. The safety of the treatment was assessed regularly by physical and blood examinations. The efficacy of Pex for preventing heterotopic ossifications was evaluated by the presence of flare-ups, measurements of serum bone markers, and changes in the total bone volume calculated by the three-dimensional computed tomography (3D-CT) images.

**Results:**

Five patients with an average age of 23.4 years were enrolled. Within safe doses of Pex administration in each individual, there were no drug-induced adverse effects during the medication phase. Three patients showed no intense inflammatory reactions during the study period, while two patients had acute flare-ups around the hip joint without evidence of trauma during the medication phase. In addition, one of them became progressively incapable of opening her mouth over the discontinuation phase. Serum levels of alkaline phosphatase (ALP) and bone specific ALP (BAP) were significantly and synchronously increased with the occurrence of flare-ups. Volumetric 3D-CT analysis demonstrated a significant increase in the total bone volume of Case 2 (378 cm^3^) and Case 3 (833 cm^3^) during the two-year study period.

**Conclusions:**

We could not prove the efficacy of oral Pex administration in the prevention of heterotopic ossifications in FOP. Serum levels of ALP and BAP appear to be promising biomarkers for monitoring the development of ectopic ossifications and efficacy of the therapy. Quantification of change in the total bone volume by whole body CT scanning could be a reliable evaluation tool for disease progression in forthcoming clinical trials of FOP.

## Background

Fibrodysplasia ossificans progressiva (FOP) (OMIM: 135100) is a severely disabling heritable disorder of connective tissue characterized by congenital malformations of the great toes and progressive heterotopic ossification in various extraskeletal sites. FOP is very rare with a worldwide prevalence of approximately 1/2,000,000 [1]. It is caused by a recurrent activating mutation (617G > A, R206H) in the gene encoding activin A receptor type I (*ACVR1*)/activin-like kinase 2 (*ALK2*), a bone morphogenetic protein (BMP) type I receptor [2]. In FOP, the mutant receptor causes up-regulation of a transcriptional factor, *Id1*. Typically, during the first decade of life, sporadic episodes of painful soft tissue swellings (flare-ups) occur, which can transform skeletal muscles, tendons, ligaments, fascia, and aponeuroses into heterotopic bone [3]. Progressive heterotopic ossifications span the joints, lock them in place, and render movement impossible [4]. Immobility is cumulative and most patients are wheelchair-bound by the end of second decade of life [5]. Attempts to remove heterotopic bones usually lead to explosive new bone formation.

At present, there is no definitive pharmacotherapy to prevent progressive heterotopic ossifications in FOP. Recently, dorsomorphin and LDN-193189, a selective inhibitor of BMP type I receptor kinases, have been reported to inhibit activation of the BMP signaling in cultures cells and mice [6,7]. Similarly, CD1530, an agonist of nuclear retinoic acid receptor-γ, prevented heterotopic ossification in FOP model mice [8]. None of these compounds, however, has been applied in clinical practice.

A promising alternative for orphan diseases is the drug repositioning strategy, in which a drug currently used for patients with a specific disease is applied to another disease [9]. The advantage of this strategy is that the identified drugs are readily available and the adverse effects are known. In order to search for clinically applicable drugs for FOP, we screened 1040 FDA-approved drugs for suppression of the *Id1* promoter activated by the mutant *ACVR1/ALK2* in mouse C2C12 myoblasts. We found that perhexiline maleate (Pex), which is a prophylactic antianginal drug widely used for stable angina but its use markedly declined in the early 1980s after reports of hepatotoxicity and peripheral neuropathy, suppressed the *Id1* promoter activity and mRNA expression of native *Id1* and alkaline phosphatase by down-regulating phosphorylation of Smad1/5/8. Pex also reduced the volume of heterotopic ossification in crude BMP-induced model mice [10]. Here, we conducted an open-labeled clinical trial of Pex administration in the management of FOP.

## Methods

This study was a non-randomized, non-placebo-controlled investigation to prospectively estimate the effect of Pex treatment in FOP patients. Eligible for participation were the patients who presented classic features of FOP including congenital malformation of the great toes and progressive heterotopic ossification of soft tissues, and those who had R206H mutation in the *ACVR1/ALK2* gene [11]. Because safety of Pex administration in children has not been established, skeletally immature patients were excluded from the study. Since there is no known effective treatment in preventing heterotopic ossification of FOP, we did not exclude the patients who received concurrent use of other medications, such as nonsteroidal anti-inflammatory drugs (NSAIDs) or cyclooxygenase-2 (COX-2) inhibitors. After approval from the Institutional Review Boards of the Nagoya University, patients who provided written informed consent were enrolled in the study.

All patients continued to receive Pex administration for a total of 12 months. At the end of this period, they discontinued Pex pharmacotherapy and were monitored for 12 consecutive months of discontinuation follow-up phase. After two weeks administration of an initial dose of 100 mg/day, plasma concentration of Pex was measured to adjust the dosage in each individual. Therapeutic drug monitoring was then regularly performed during the medication phase by Drs. John D. Horowitz and Benedetta C. Sallustio (Queen Elizabeth Hospital, Woodville, Australia), and an optimal dose of oral Pex administration was individually determined based on a range for Pex of 0.15-0.60 mg/L. The Safety of treatment was assessed by a monthly physical examination and a complete blood count/serum chemistry evaluation every three months, with a special care for known adverse effects of Pex including peripheral neuropathy and drug induced hepatic dysfunction [12]. The efficacy of Pex for preventing heterotopic ossifications was evaluated clinically and biochemically, as well as by volumetric computed tomography (CT). Careful physical examination was performed on each patient to observe the presence of flare-ups and the development of new ectopic ossifications. Serum concentrations of non-specific alkaline phosphatase (ALP), bone-specific alkaline phosphatase (BAP) and osteocalcin (OC) were measured at baseline, after 1, 3, 6, 9, and 12 months of Pex treatment (M: medication phase), and after 1, 3, 6, 9, and 12 months of medication discontinuation (D: discontinuation phase), using the commercially available Japan Society of Clinical Chemistry (JSCC) method, enzyme immunoassay (EIA), and radioimmunoassay (RIA) respectively (SRL Inc, Japan). For quantitative evaluation of ectopic bones to be formed, whole body scanning by 16 slice multi-detector CT was performed before the intervention (baseline), at the end of Pex medication (M-12 m: 12 months after commencement of treatment), and at the end of the study (D-12 m: 12 months after medication discontinuation). Due to various degrees of contractures in the upper and lower extremities as well as in the trunk, the top of the skull or periphery of the limbs sometimes failed to be imaged in some patients. Thus, we defined structural regions of interest (ROI) as the maximum 3D-CT imaging ranges to be analyzable which was standardized in each individual. Based on 3D-CT images, total bone volume (expressed as cm^3^) in each patient was calculated by quantitative density analysis. The volume of newly formed bones was quantified by change in the total bone volume during the medication and discontinuation phases.

## Result

Five FOP patients were enrolled in the study between July 2010 and July 2012 (Table 1). There were three males and two females with an average age of 23.4 years (range, 18–36 years). All patients had significant deformities associated with severely restricted mobility of the spine and limbs. Two patients were confined to a wheelchair and required assistance in performing activities of daily living. Two patients received concurrent treatment with COX-2 inhibitor on a regular basis, and three patients irregularly took fast-acting NSAIDs when they felt pain. Under strictly controlling plasma concentration of Pex within 0.15-0.60 mg/L, the steady dosage of Pex varied between individuals from 14 mg/day (100 mg/week) to 200 mg/day. No obvious drug-induced adverse effects were found and no patients discontinued Pex administration during the whole period of treatment.

**Table 1:** Patients’ characteristics and clinical outcome

**Case**	**Age (years)**	**Gender**	**Dose of Pex**	**Adverse events**	**Acute inflammatory reaction (site)**
1	36	Male	150 mg/d	None	None
2	26	Male	200 mg/d	None	M-7 m (right proximal thigh)
3	18	Female	75 mg/d	None	M-8 m (left hip), D-2 m (right jaw)
4	18	Female	14 mg/d	None	None
5	19	Male	100 mg/d	None	None

Pex = Perhexiline maleate; M-7 m = Medication phase at 7 months.

M-8 m = Medication phase at 8 months; D-2 m = Discontinuation phase at 2 months.

In three of the five patients, there were no intense inflammatory reactions associated with flare-ups during the study period, although this could happen randomly (Table 1). On the other hand, acute flare-ups were observed in two patients (Cases 2 and 3) without evidence of trauma during the medication phase (M-7 m and M-8 m, respectively) and high-dose corticosteroid treatment was administered in each patient at the beginning of flare-ups according to the treatment guidelines of International Fibrodysplasia Ossificans Progressiva Association (IFOPA) [13]. These flare-ups occurred in the right proximal thigh in Case 2 and around the left hip joint in Case 3. Following flare-ups, their hip joint mobility gradually deteriorated. In addition, Case 3 complained of severe right jaw pain and subsequent difficulty in mouth opening during the early discontinuation phase (D-2 m). Limited opening of the mouth resulted in interference with eating and oral hygiene.

**Figure 1:**
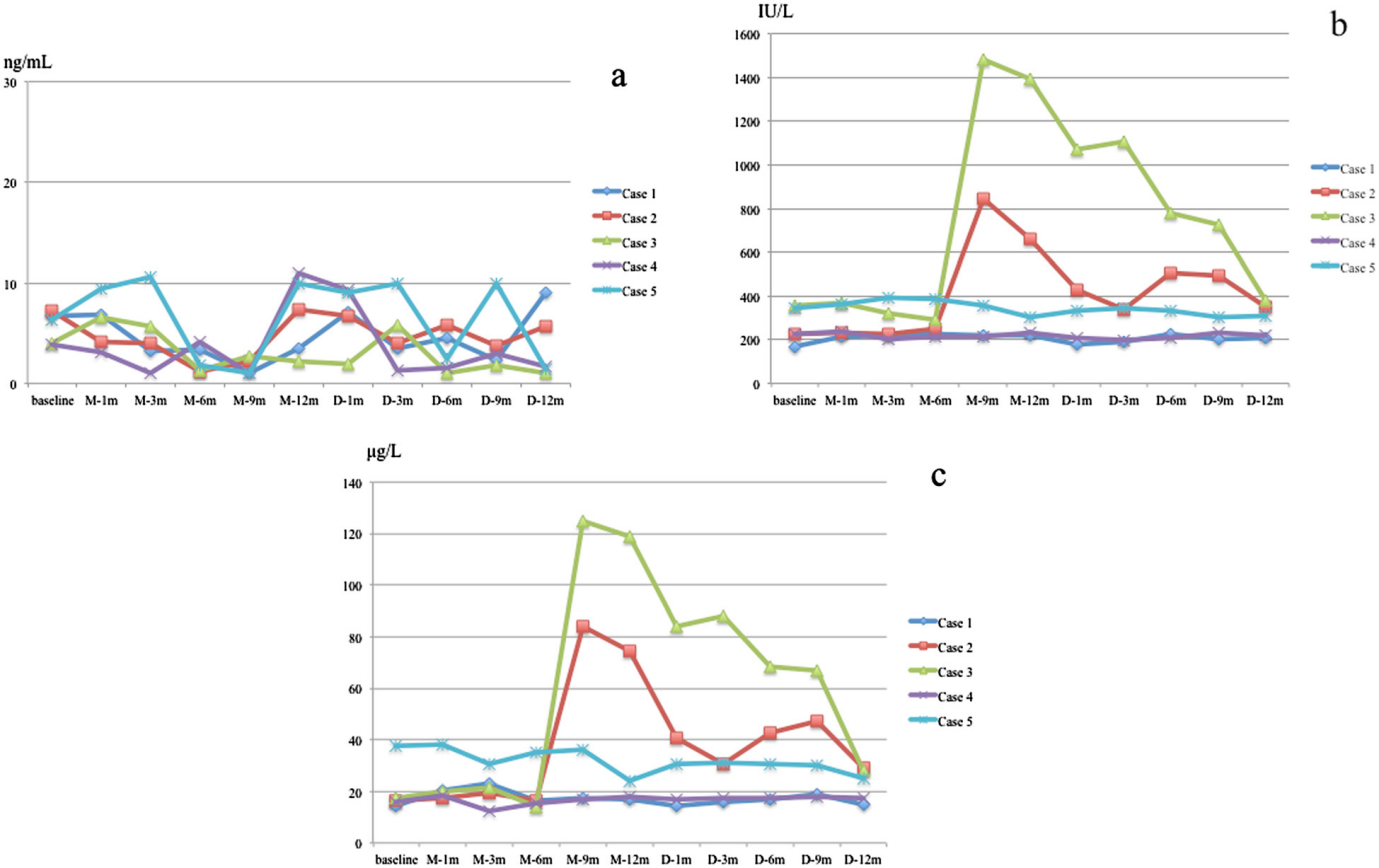
**Serial measurements of serum osteocalcin (a), alkaline phosphatase (ALP) (b) and bone-specific alkaline phosphatase (BAP) (c) from 5 FOP patients.** Serum levels of osteocalcin were maintained within the normal range during the study period in all patients. Serum levels of ALP rapidly elevated at M-9 m in Cases 2 and 3 who showed acute flare-ups at M-7 m and M-8 m, respectively. Case 3, who showed acute inflammation of her right jaw at D-2 m, had persistent higher ALP levels at D-3 m. Changes in serum BAP activity positively correlated to those in serum ALP levels.

Serum concentration of OC had no significant change in all patients (Figure 1a). During the whole study period, serum ALP and BAP levels were maintained at a normal range in the three patients who did not have inflammatory reactions. In the other two patients (Cases 2 and 3), on the other hand, these bone markers significantly and synchronously elevated following the occurrence of flare-ups during medication phase (Figure 1b,c). Elevated ALP and BAP levels were gradually reduced with time, but in Case 3, both bone markers rebounded with acute inflammation of her right jaw during the early discontinuation phase.

**Figure 2:**
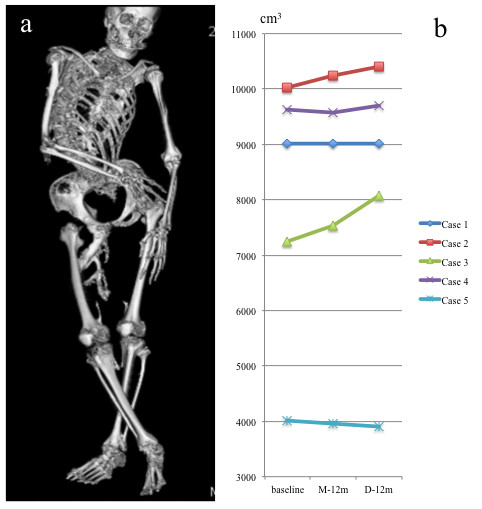
**Volumetric computed tomography (CT) examination.** Representative reconstructed 3D-CT image obtained by whole body scanning of Case 1 **(a)**, and changes in total bone volume within the region of interests (ROI) in each FOP patient **(b)**. In Case 2, total bone volume has increased 223 cm^3^ and 155 cm^3^ during the medication and discontinuation phases, respectively. In Case 3, there was an increase of 297 cm^3^ and 536 cm^3^ in the total bone volume during the medication and discontinuation phases, respectively.

**Figure 3:**
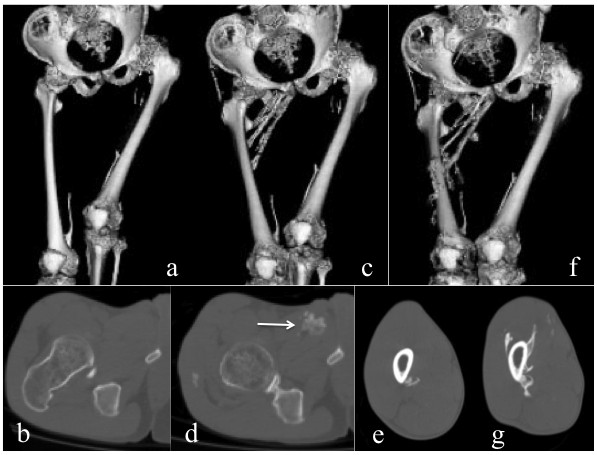
**CT images from Case 2.** Reconstructed 3D-CT images of the pelvis and bilateral femora at baseline **(a)**, at M-12 m **(c)**, and at D-12 m **(f)**. Axial CT images of the right hip at baseline **(b)**, and at M-12 m **(d)**, and those of the right mid-femur at M-12 m **(e)**, and at D-12 m **(g)**. Heterotopic ossifications in the right adductor muscle (arrow) developed during the medication phase and around mid-femur developed during the discontinuation phase ware clearly demonstrated.

**Figure 4:**
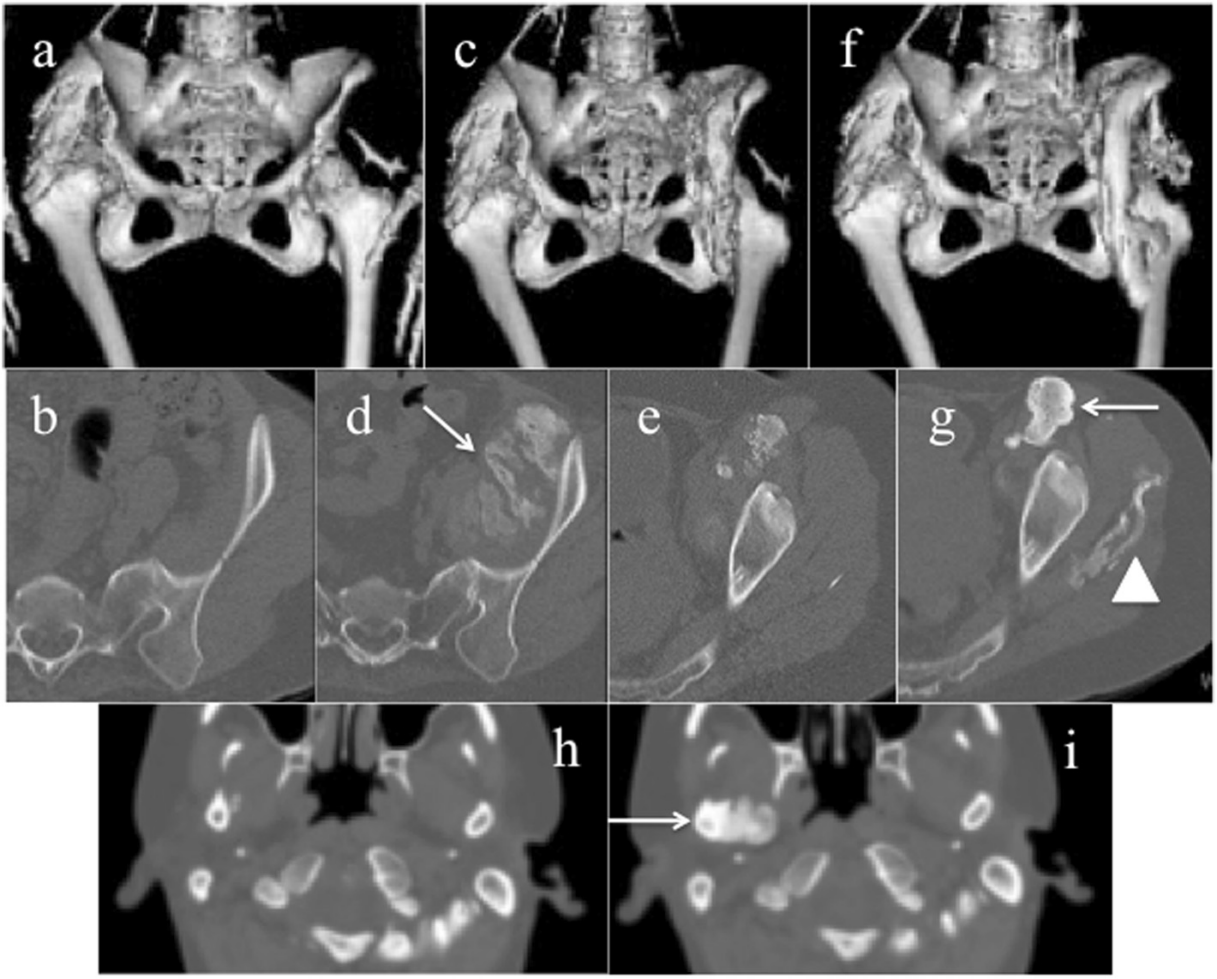
**CT images form Case 3.** Reconstructed 3D-CT images of the hip joints at baseline **(a)**, at M-12 m **(c)**, and at D-12 m **(f)**. Axial CT images of the left pelvis at baseline **(b)**, at M-12 m **(d**, **e)**, and at D-12 m **(g)**, and those of the bilateral jaws at M-12 m **(h)**, and at D-12 m **(i)**. Massive new bone formations in the left iliac muscle were developed during the medication phase **(d**, arrow**)**. During the discontinuation phase, the left intra-iliopsoas ossification matured **(g**, arrow**)**, and heterotopic boned in the left gluteus medius **(g**, arrow head**)** and the right jaw joint **(i**, arrow**)** were newly formed.

Quantitative 3D-CT analysis demonstrated that the total bone volume did not change in three patients (Cases 1, 4, and 5) during the study period, while a substantial increase in the total bone volume, both during the medication and discontinuation phases, was found in two patients (Figure 2a,b). In Case 2, increased bone volume of 223 cm^3^ during the medication phase and that of 155 cm^3^ during the discontinuation phase seemed to be associated with heterotopic bone formations in the right adductor muscle, and around the mid-femur, respectively (Figure 3a-g). In Case 3, there was an increase of 297 cm^3^ in the total bone volume during the medication phase, which seemed to be related to intramuscular ossification in her left iliopsoas (Figure 4a-d). She also showed a maximal increased bone volume of 536 cm^3^ during the discontinuation phase, which appeared to reflect maturation of the iliopsoas ossification and newly developed bones in the gluteus medius (Figure 4e-g) and around the right jaw joints (Figure 4h,i).

## Discussion

To date, there are few clinical trials in the treatment of FOP. Zasloff et al. [14] conducted a prospective study to assess the efficacy of isotretinoin (13-cis-retinoic acid) in the prevention of heterotopic ossification in FOP, and concluded that isotretinoin had no apparent effect in the prevention of new bone formation after surgery or after soft tissue trauma. Brantus and Meunier [15] evaluated the effects of intravenous administration of etidronate and oral corticosteroids for thirty-one FOP attacks in seven patients, and observed 10 new ossifications causing severe deterioration of joint mobility during the mean six years of follow up. These studies indicated that there is no proven efficacy with any therapy in changing the natural history of the disease. Despite the Pex treatment, heterotopic ossification developed rapidly in our two patients suggesting that oral administration of Pex within 0.15-0.60 mg/L seemed to be unsatisfactory in the inhibition of heterotopic ossifications in FOP. Moreover, there is a concerning possibility that Pex administration unexpectedly induced heterotopic ossification in these patients.

Abnormal biochemical measurements of bone mineral metabolism have rarely been reported in FOP [16]. Establishment of useful biomarkers as correlates of disease severity and clinical outcome is desirable to enable early proof-of-concept studies that can help screen potential drug candidates and identify therapeutic targets. Kaplan et al. [17] described that serum ALP activity may increase during disease flare-ups. Our serial clinical and biochemical evaluations demonstrated that elevation of serum ALP and BAP, which synchronized with acute flare-ups, preceded the heterotopic new bone formations. Serum levels of ALP or BAP could be useful biomarkers for monitoring the development of heterotopic ossifications and efficacy of the therapy in FOP.

Multi-detector-row CT has widely been used in clinical environments, and whole body scanning allows 3D structural characterization of entire bone segment at high resolution [18]. Despite the restricted movement and joint immobilization of our patients, standardization of ROI in each individual could allow for accurate volume calculating capabilities. To the best of our knowledge, no studies have presented quantitative assessment of ectopic bone formations in FOP. Volumetric 3D-CT analyses demonstrated that change in the total bone volume correlated with the clinical symptoms and laboratory examinations in two patients who showed active flare-ups. Our study highlights greater capabilities of whole body CT scanning as an evaluation tool for disease progression in FOP, especially in assessment of treatment efficacy during forthcoming clinical trials.

There are several major limitations in the present study. First, since there are no better natural history studies for FOP to date, it is difficult to design any clinical trial with meaningful endpoints. Furthermore, we do not know the natural evolution of ectopic bone formations in our patients. Second, the present study could not be designed for pediatric FOP patients because of uncertainty in safety, tolerability, and pharmacokinetics of Pex in the pediatric population. Third, heterotopic ossification in FOP is generally formed via an endochondral ossification process, but we did not confirm heterotopic cartilage formation in our patients. Quantification of total bone volume based on 3D-CT images could be a reliable evaluation tool for assessment of ectopic bone formations, but radiation exposure by CT examination may be a major issue for young patients. Besides, whole body scanning has always been uncomfortable for severely deformed FOP patients, although it takes less than five minutes. Without scanning the whole body, heterotopic ossification following flare-ups (if they occur) could be evaluated by a narrow scan around the flare-ups region. For future clinical trials, standardization of imaging protocol will be expected in evaluating heterotopic bones in FOP.

## Conclusions

Although the number of patients is too small to draw reliable conclusions, oral administration of Pex within the safety dose seemed not to be effective in the inhibition of heterotopic ossifications in FOP, despite the absence of significant adverse effects.

## Abbreviations

FOP: Fibrodysplasia ossificans progressiva; ACVR1: Activin A receptor type I; ALK2: Activin-like kinase 2; BMP: Bone morphogenetic protein; Pex: Perhexiline maleate; NSAIDs: Nonsteroidal anti-inflammatory drugs; COX-2: Cyclooxygenase-2; CT: Computed tomography; ALP: Alkaline phosphatase; BAP: Bone-specific alkaline phosphatase; OC: Osteocalcin; JSCC: Japan society of clinical chemistry; EIA: Enzyme immunoassay; RIA: Radioimmunoassay; 3D: Three-dimensional; ROI: Regions of interest; IFOPA: International Fibrodysplasia ossificans progressiva association.

## Competing interests

The authors declare that they have no competing interests.

## Authors’ contribution

H Kitoh did most of the patients’ follow up, participated in the whole study and drafted the manuscript. MA did the CT interpretations. H Kaneko, MK, MM, IK participated in the clinical trial. JDH and BCS measured plasma concentration of Pex and suggested an optimal dose of Pex administration. KO and NI participated in the design of the study. All authors contributed to elaborating the manuscript. All authors read and approved the final manuscript.

## Authors’ information

H Kitoh is a member of the Japanese Research Committee on Fibrodysplasia Ossificans Progressiva.
